# Serum Profiles of C-Reactive Protein, Interleukin-8, and Tumor Necrosis Factor-*α* in Patients with Acute Pancreatitis

**DOI:** 10.1155/2009/878490

**Published:** 2010-01-14

**Authors:** Michael K. Digalakis, Iraklis E. Katsoulis, Kalliopi Biliri, Katina Themeli-Digalaki

**Affiliations:** ^1^Department of Surgery, “Asklepieio Voulas” General Hospital, 16673 Athens, Greece; ^2^Department of Microbiology, “Tzaneio” General Hospital, 18536 Piraeus, Greece

## Abstract

*Background-Aims*. Early prediction of the severity of acute pancreatitis would lead to prompt intensive treatment resulting in improvement of the outcome. The present study investigated the use of C-reactive protein (CRP), interleukin IL-8 and tumor necrosis factor-*α* (TNF-*α*) as prognosticators of the severity of the disease. *Methods*. Twenty-six patients with acute pancreatitis were studied. Patients with APACHE II score of 9 or more formed the severe group, while the mild group consisted of patients with APACHE II score of less than 9. Serum samples for measurement of CRP, IL-8 and TNF-*α* were collected on the day of admission and additionally on the 2nd, 3rd and 7th days. *Results*. Significantly higher levels of IL-8 were found in patients with severe acute pancreatitis compared to those with mild disease especially at the 2nd and 3rd days (*P* = .001 and *P* = .014, resp.). No significant difference for CRP and TNF-*α* was observed between the two groups. The optimal cut-offs for IL-8 in order to discriminate severe from mild disease at the 2nd and 3rd days were 25.4 pg/mL and 14.5 pg/mL, respectively. *Conclusions*. IL-8 in early phase of acute pancreatitis is superior marker compared to CRP and TNF-*α* for distinguishing patients with severe disease.

## 1. Introduction

Acute pancreatitis remains a common disease in western societies and in most cases it resolves spontaneously with supportive treatment. Nevertheless, 25% of the patients will develop local or systemic complications which may require surgical intervention. The most crucial point is the development of pancreatic necrosis, which often progresses to a systemic inflammatory response syndrome (SIRS). An approximate mortality of 10%–30% has been reported in patients with severe forms of acute pancreatitis while most deaths are attributed to sepsis and to multiple organ dysfunction syndrome (MODS).

Staging the severity of acute pancreatitis based on clinical parameters has limited specificity and sensitivity for the prediction of adverse events. Already from the decade of 1980s staging using serum criteria was employed [1–3].

As an inflammatory process, acute pancreatitis results in an excessive leukocyte activation and increased migration of neutrophils to the inflamed area with a consequent release of proinflammatory mediators including interleukins (IL-1b, IL-6, IL-8, IL-10, IL-18) and Tumor Necrosis Factor-alpha (TNF-*α*) [4–11]. These mediators have been involved with the pathogenesis of progression of a pancreatic infection to necrosis and consequently to SIRS and multiorgan failure.

The detection of cytokines appears to provide a more accurate and objective method for the assessment of the severity of acute pancreatitis [4, 5, 8, 9, 11]. Many clinical trials have employed the use of either cytokines [4, 5, 8, 9, 11] or serum amyloid A and procalcitonin [11–15] as prognosticators of the severity of acute pancreatitis.

Recent studies have suggested that the serum levels of interleukins and TNF-*α* may be used to identify patients who are prone to develop local or systemic complications and were compared with CRP which has been employed in the prediction of severity of acute pancreatitis [4, 7–11]. Early identification of such patients could lead to a more intensive management that would result to a decreased morbidity and mortality of that potentially fatal disease [4, 7–11].

The present study aimed to assess Interleukin-8 (IL-8), C-reactive protein, and tumor necrosis factor-*α* as prognosticators of a severe course of acute pancreatitis.

## 2. Patients and Methods

Twenty-six patients who were admitted to hospital with the diagnosis of acute pancreatitis were evaluated during the second semester of 2007. These were 15 men and 11 women with a mean age of 66 years. Patients with presentation more than 24 hours from the onset of symptoms, pancreatic tumor, prior pancreatic surgery, renal or hepatic failure, trauma and diabetic ketoacidocis were excluded from the study.

The diagnosis was based on the presenting signs and symptoms (acute abdominal pain, vomiting, etc.), the elevation of serum and urine amylase (>1000 IU/L), and the findings of the upper abdominal ultrasonography. The confirmation of diagnosis was made by Computed Tomography with contrast within 72 hours from the time of the admission.

All patients were evaluated according to Acute Physiology and Chronic Health Evaluation II (APACHE II) score determined within 48 hours of admission [16]. Those patients with a score of 9 or more formed the severe group while the rest formed the mild group. The identification of the aetiology of acute pancreatits was based on medical history and on laboratory and imaging evaluation (Table 1).

Serum samples of both interleukin 8 (IL-8) C-reactive protein and Tumor necrosis factor-*α* were collected on the day of admission and additionally on the 2nd, 3rd, and 7th days and they were stored at −80°C [5, 8]. Serum levels of IL-8 and TNF-*α* were examined with enzyme-linked immunosorbent assay (ELISA) using kits (Anibion, Orgenium Laboratories, Finland). CRP was determined with nephelometric technique (Dade Behring Inc).

IL-8, TNF-*α*, and CRP levels are presented as median values (interquartile range). For the comparison of IL-8, TNF-*α*, and CRP levels between the two groups Mann-Whitney *U* test was used. IL-8, TNF-*α*, and CRP were tested for their ability to discriminate between mild and severe group using receiver operating characteristic (ROC) curves. The overall performance of the ROC analysis was quantified by computing area under the curve (AUC) and 95% confidence intervals. An area of 1 indicated perfect performance, while 0.5 indicated a performance that was not different than chance. Using ROC analysis were determined the optimal sensitivity and specificity of using various cut-off values for the discrimination between the two groups. Sensitivity, specificity, positive and negative predictive values are presented for the results of ROC analyses concerning optimal cut-offs. The reported *P*-values are two-tailed. Statistical significance was set at 0.05, and analysis was conducted using STATA 7.0.

All the patients were closely followed according to the Atlanta criteria [17], for the development of local (pancreatic collection, ascites, necrosis, and pseudocyst formation) or systemic (ARDS, MODS, septic shock) complications and managed accordingly (ERCP, CT-guided drainage, surgery).

## 3. Results

**Figure 1:**
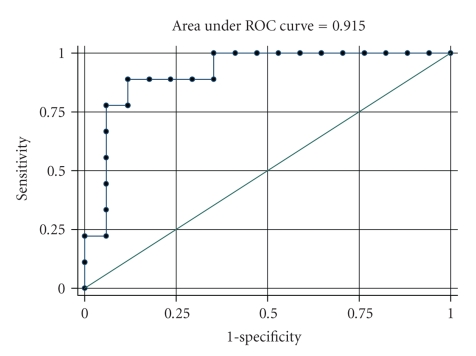
ROC curve for the discrimination between mild and severe groups from IL-8 at second day.

**Figure 2:**
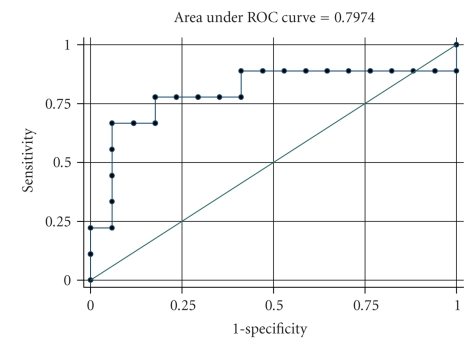
ROC curve for the discrimination between mild and severe groups from IL-8 at third day.

The median values of IL-8, TNF-*α*, and CRP levels from the first to the seventh days for the two groups are presented in Table 2. The median value of IL-8 at the second day was 53.2 pg/mL (range: 37.8–61) for the severe group and 12.9 pg/mL (range: 9.4–20) for the mild group, while the median value of IL-8 at the third day was 37.3 pg/mL (range: 25.1–60.6) for the severe group and 11.6 pg/mL (range: 6.8–19.2) for the mild one. IL-8 levels were significantly greater for severe group at the second and third day (*P* = .001 and *P* = .014, resp.). TNF-*α* and CRP levels were not different between the two groups at any day, indicating no prognostic ability to discriminate between the two groups. ROC analysis was used for IL-8 in order to find the optimal cut-off for the discrimination of severe form mild group (Table 3). The AUC for IL-8 at second and third day was 0.92 and 0.80, respectively, (Figures 1 and 2). The optimal cut-off for IL-8 in order to discriminate severe from mild disease at the second day was 25.4 pg/mL with sensitivity equal to 88.9% and specificity equal to 88.2%. Additionally, the optimal cut-off for IL-8 at the third day was 14.5 pg/mL with sensitivity equal to 88.9% and specificity equal to 55.8%.

## 4. Discussion

Interleukin-8 (IL-8) is one of the most important inflammatory mediators in acute pancreatitis and other inflammatory processes. IL-8 is released by many cell lines in the presence of activated neutrophils [18–21]. It is also considered to be one of the main secondary mediators of TNF-*α* induced neutrophil activation [22]. On the other hand, TNF-*α* which is a pleiotropic predominantly macrophage-derived cytokine is thought to be involved in pathophysiological responses in cases of severe injury or sepsis [20, 22]. C-reactive protein (CRP) that is synthesized by the hepatocytes is a nonspecific inflammatory marker routinely used in assessment of severity of acute pancreatitis [18, 20, 22]. Its synthesis is induced by the release of interleukins 1 and 6 thus a serum peak is usually delayed (>3rd day of onset of pain) [18, 20, 22].

Acute pancreatitis, regardless its aetiology, leads to the disruption of the normal stimulation-secretion axis within the pancreatic acinar cell. This ultimately triggers a premature enzyme activation system (concerning mainly the metabolism of trypsinogen to trypsin) and a consequent cascade of coactivation of other pancreatic proenzymes (such as proelastase, chymotrypsinogen, procarboxypeptidase, and phosholipase A2), causing autodigestion of the gland [18, 19, 22, 23]. Blood monocytes and neutrophiles migrate to the site of inflammation and secrete inflammatory mediators [18].

In cases of acute pancreatitis with limited extent of injury, spontaneous resolution of the inflammation occurs. When inflammation persists, mediators are released to the circulation leading to the malfunction of various organs and a systematic inflammatory response (SIRS) [18, 19, 23]. This progression from acute pancreatic inflammation to SIRS and MODS may often be fulminant as approximately 50–60% of severe acute pancreatitis deaths occur within the first week [18, 20]. This systemic reaction combined with local complications such as fluid collection, haemorrhage, and necrosis necessitates an early prediction in order to achieve a more aggressive treatment. Most of the deaths reported after the first weeks are due to secondary pancreatic infection and necrosis [12, 18]. Some authors support that systematic inflammatory response may occur even in the absence of pancreatic necrosis [7].

Already from the decade of 1980s staging of the severity of acute pancreatitis using serum criteria was employed [1–3]. Many clinical trials have employed the use of either cytokines [4, 5, 8, 9, 11] or serum amyloid A and procalcitonin (PCT) [11–15] as prognosticators of the severity of acute pancreatitis. Many authors suggest that PCT may accurately predict infected pancreatic necrosis. Other studies over the last few years have suggested that serum levels of interleukins and TNF-*α* may be used to identify patients who are prone to develop local or systemic complications and were compared with CRP which has been employed in the prediction of severity of acute pancreatitis. [4, 7–11]. Early identification of such patients could lead to a more intensive management that would result to a decreased morbidity and mortality of that potentially fatal disease [4, 7–11].

In the present study, we identified significantly higher serum levels of IL-8 in patients with severe acute pancreatitis, compared to patients with mild or moderate disease. On admission, mean plasma levels of neither IL-8 nor TNF-*α* and CRP showed any significant difference between two groups but on day 2, significantly higher values for IL-8 were observed on the group of patients with severe pancreatitis. On the third day, the statistical difference between the two groups was continued for the IL-8 levels. TNF-*α* and CRP levels did not show any significant difference between the two groups even in the latest measurement (7th day). In all patients of the mild group resolution occurred with supportive treatment within less than one week and no local or systemic complications were identified. On the contrary, the majority of patients in the severe group developed several either serious (ARDS, MODS, septic shock, pancreatic necrosis) or less important complications (3 patients developed only mild peripancreatic fluid collections without the evidence of a pseudocyst formation). Three patients were transferred to the ICU due to multiorgan dysfunction and one of them who underwent multiple surgical interventions due to recurrent pancreatic necrosis eventually died.

The highest sensitivity and diagnostic accuracy for IL-8 was observed on the second day. This statistically significant difference suggests that serum IL-8 levels may be a superior marker for the early prediction of severe disease comparing to clinical criteria which have a limited prognostic value.

We believe that the persistence of high levels of IL-8 levels during the first week of severe acute pancreatitis may be related to the development of late local and systemic complications or its progression to chronic pancreatitis. TNF-*α* is known to stimulate the production of several cytokines including IL-8 and propagate the cascade of phenomena in severe sepsis [4]. Nevertheless, our study did not demonstrate a role of TNF-*α* as a predictive marker of the severity of acute pancreatitis. Moreover, although CRP was elevated in patients with extensive pancreatic necrosis with bacterial colonization and abscess formation, it did not differ significantly from mild cases.

The aim of our study was to investigate the correlation of cytokines with the severity and prognosis of acute pancreatitis. Our findings suggest the pivotal role of interleukins to the progression of pancreatic injury to systemic inflammation.

Since the development of multiorgan failure decreases dramatically the prognosis in acute pancreatitis patients, it is of paramount importance to promptly identify high-risk patients. Measurement and followup of IL-8 serum levels seems to be an accurate method in order to assess the extent and persistence of the inflammatory process that can contribute to an early and more accurate management of this fragile patient group.

## Figures and Tables

**Table 1:** Aetiology of acute pancreatitis (*n* = 26).

Aetiology	Female (*n* = 11)	Male (*n* = 15)	Mild (*n* = 17)	Severe (*n* = 9)
Gallstones	9	5	11	3
Alcohol	1	7	4	4
Other	1	3	2	2

**Table 2:** IL-8, TNF-*α*, and CRP levels for mild and severe groups.

IL-8	Mild (*n* = 17)	Severe (*n* = 9)	
	Median (Int. range)	Median (Int. range)	P*
1st day	24.5(10.3–37.5)	71(14.5–160)	.124
2nd day	12.9(9.4–20)	53.2(37.8–61)	.001
3rd day	11.6(6.8–19.2)	37.3(25.1–60.6)	.014
7th day	11.5(5.1–22.2)	21.5(12.3–120)	.063

TNF-alpha			
TNF-*α* (1st day)	2(1–4.2)	2(1–11.3)	.423
TNF-*α* (2nd day)	2(1–4.1)	1(1–10.5)	.757
TNF-*α* (3rd day)	2(1–4.1)	1(1–24.5)	.933
TNF-*α* (7th day)	2(1–4.1)	1(1–14.5)	.609

CRP			
CRP (1st day)	28.7(4.4–66)	30.5(18.6–36)	.389
CRP (2nd day)	92.6(50.1–121)	119(107–170)	.189
CRP (3rd day)	116(59.4–154)	161(144–196)	.130
CRP (7th day)	23.9(6.5–87)	56(27–79)	.305

*Mann-Whitney *U* test.

**Table 3:** Results from the ROC analysis of IL-8 for the discrimination between mild and severe groups.

	AUC (95% CI)	*P*	Optimal cut-off	Sensitivity %	Specificity %	PPV	NPV
IL-8 (2nd day)	0.92(0.80–1.00)	.001	25.4	88.9	88.2	80.0	93.8
IL-8 (3rd day)	0.80(0.58–1.00)	.014	14.5	88.9	55.8	53.3	90.9

AUC: Area Under the Curve; CI: Confidence Intervals; PPV: Positive Predictive Value; NPV: Negative Predictive Value.
